# uPA/uPAR and SERPINE1 in head and neck cancer: role in tumor resistance, metastasis, prognosis and therapy

**DOI:** 10.18632/oncotarget.10344

**Published:** 2016-06-30

**Authors:** Miguel Angel Pavón, Irene Arroyo-Solera, Maria Virtudes Céspedes, Isolda Casanova, Xavier León, Ramón Mangues

**Affiliations:** ^1^ Grupo d’Oncogènesi i Antitumorals, lnstitut d’Investigacions Biomèdiques Sant Pau (IIB-Sant Pau), Barcelona, Spain; ^2^ Infections and Cancer Unit, Cancer Epidemiology Research Programme, Catalan Institute of Oncology (ICO) and Bellvitge Institute of Biomedical Research (IDIBELL), Barcelona, Spain; ^3^ Centro de Investigación Biomédica en Red en Bioingeniería, Biomateriales y Nanomecidicina (CIBER-BBN), Barcelona, Spain; ^4^ Department of Otorrinolaryngology, Hospital de la Santa Creu i Sant Pau, Barcelona, Spain

**Keywords:** head and neck cancer, uPA, uPAR, SERPINE1, prognosis

## Abstract

There is strong evidence supporting the role of the plasminogen activator system in head and neck squamous cell carcinoma (HNSCC), particularly of its uPA (urokinase plasminogen activator) / uPAR (urokinase plasminogen activator receptor) and SERPINE1 components. Overexpression of uPA/uPAR and SERPINE1 enhances tumor cell migration and invasion and plays a key role in metastasis development, conferring poor prognosis. The apparent paradox of uPA/uPAR and its inhibitor SERPINE1 producing similar effects is solved by the identification of SERPINE1 activated signaling pathways independent of uPA inhibition. Both uPA/uPAR and SERPINE1 are directly linked to the induction of epithelial-to-mesenchymal transition, the acquisition of stem cell properties and resistance to antitumor agents. The aim of this review is to provide insight on the deregulation of these proteins in all these processes.

We also summarize their potential value as prognostic biomarkers or potential drug targets in HNSCC patients. Concomitant overexpression of uPA/uPAR and SERPINE1 is associated with a higher risk of metastasis and could be used to identify patients that would benefit from an adjuvant treatment. In the future, the specific inhibitors of uPA/uPAR and SERPINE1, which are still under development, could be used to design new therapeutic strategies in HNSCCs.

## INTRODUCTION

Head and neck cancer is the sixth most common cancer in incidence worldwide [1]. More than 500,000 new cases of head and neck squamous cell carcinoma (HNSCC) are diagnosed each year (http://globocan.iarc.fr). Two thirds of patients are diagnosed at advanced stages, as lymph node metastases are often the first sign of the disease [2]. In advanced stages, surgery can significantly impact organ function, produce damage to the structures involved in swallowing and speech, and greatly reduce patient quality of life [3]. In order to avoid these effects, initial radical surgery has been progressively replaced by multimodal treatments that combine surgery, radiation and chemotherapy. Multimodal treatments have improved loco-regional disease control and organ preservation in head and neck patients, but five-year survival remains around 50% [2]. A high percentage of patients develop recurrences, metastasis or secondary tumors after treatment, which results in a poor clinical outcome [4-8].

The molecular mechanisms associated with head and neck tumor invasion, metastasis, dissemination and drug resistance remain largely unknown. The identification of new biomarkers associated with tumor spread could be very useful in classifying patients according to their risk of recurrence [9]. An appropriate classification would make it possible to optimize and rationalize treatment, management and HNSCC patient care after diagnosis [10, 11].

**Figure 1:**
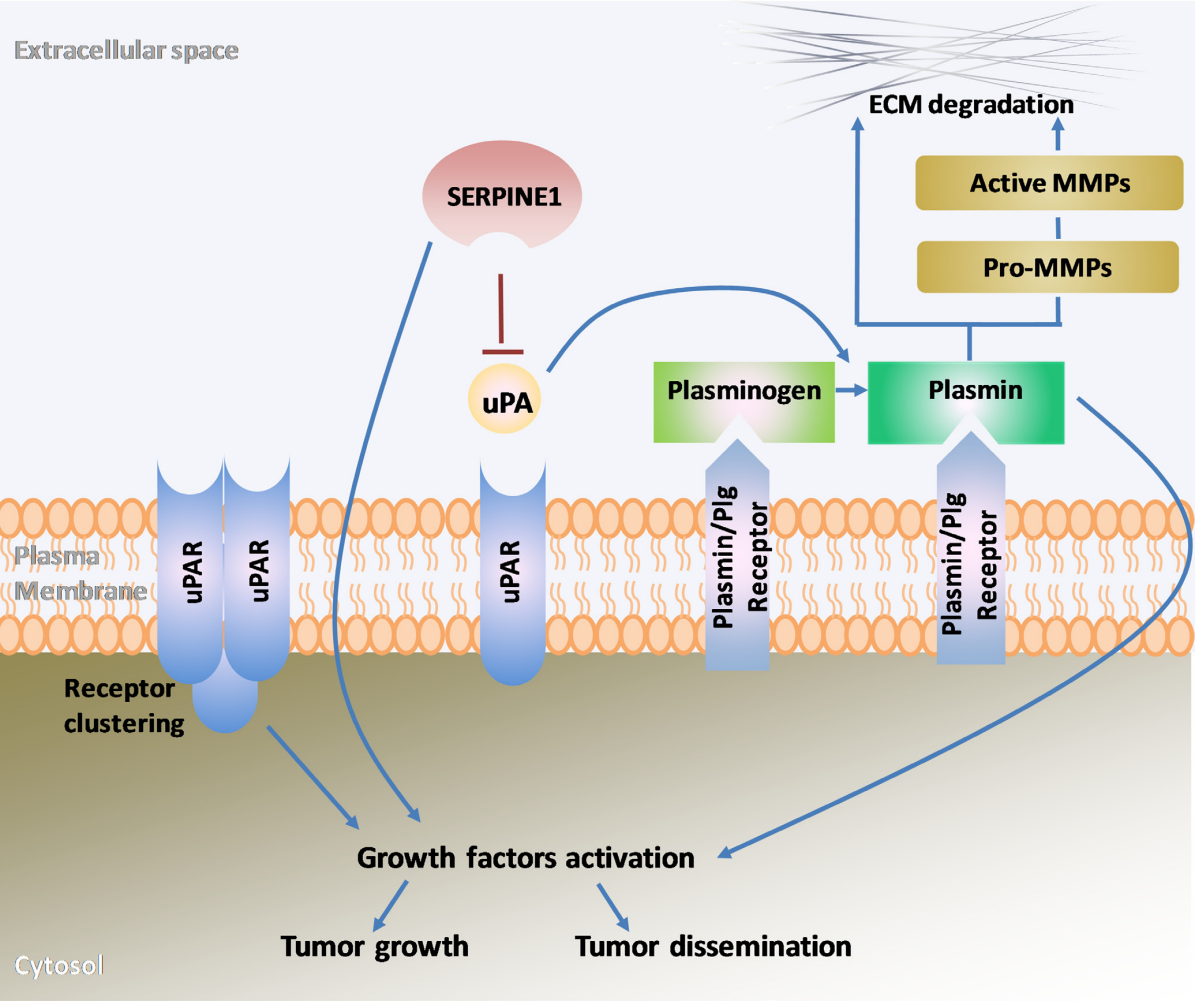
Schematic representation of the main components of the plasminogen activator system and their role in extracellular matrix remodeling, growth factor activation, tumor growth and dissemination uPA, urokinase plasminogen activator; uPAR, urokinase plasminogen activator receptor; SERPINE1, serpin family E member 1 also known as plasminogen activator inhibitor-1(PAI-1); Plg, plasminogen; MMPs, metalloproteinases; ECM, extracellular matrix.

The plasminogen activator (PA) system plays a key role in extracellular matrix (ECM) remodeling, which in turn is crucial in the first steps of tumor progression and spreading [12]. Its main components include the plasminogen activators (uPA, urokinase plasminogen activator; tPA, tissue plasminogen activator), the cell membrane receptor for uPA (uPAR), the plasminogen activator inhibitors (PAI-1, plasminogen activator inhibitor 1; PAI-2, plasminogen activator inhibitor 2) and plasmin (Figure 1) [13]. The PA system regulates the generation of plasmin that results from the activation of plasminogen by uPA or tPA. The uPAR receptor in turn accumulates plasminogen conversion at cell surfaces [12]. In addition, the plasminogen activator inhibitors (PAI-1; PA1-2), also known as, SERPINE1 and SERPINB2 are the main inhibitors of uPA and tPA [13, 14].

Plasmin activation is a key factor for fibrinolysis control and prevents health problems due to the formation of blood clots. Deregulation of uPA/uPAR and SERPINE1 has been associated with thrombosis, cardiovascular diseases and alterations of wound healing [14, 15]. Moreover, the PA system effect on cell adhesion and migration is particularly important in cancer progression. Active plasmin degrades the ECM directly or indirectly though the activation of several metalloproteinases [14]. ECM degradation facilitates the migration of tumor cells to other tissues and structures. SERPINE1 regulates this process and the adhesion/deadhesion balance of cells to the ECM, which is essential to control and promote tumor cell migration [16, 17]. A high expression of uPA/uPAR and SERPINE1 has been observed in numerous cancer types, being associated with poor patient prognosis [12, 18]. In this regard, several studies supporting the association between the activation of the PA system and head and neck cancer prognosis have been reported over the last years.

In this review, we first describe the effect of uPA, uPAR and SERPINE1 in head and neck cancer cell migration, metastatic dissemination and drug resistance. Secondly, we summarize their value as prognostic markers in patients with HNSCC. Finally, we discuss about the potential use of SERPINE1, uPAR, uPA and associated pathways as therapeutic targets.

## ROLE OF UPA/UPAR IN TUMOR CELL PROLIFERATION, MIGRATION, INVASION AND METASTASIS

The uPA gene (Gene ID: 5328; http://www.ncbi.nlm.nih.gov/) encodes a 411 amino acid serine protease that consists of two α and two anti-parallel β strands [19]. This protein is secreted as a zymogen (pro-uPA) that is activated by cleavage of the peptide bond Lys158-Ile159 [20]. uPA has an amino-terminal EGF-like domain, a kringle domain, an interdomain linker and a catalytic domain [21]. The pro-uPA to uPA conversion is catalyzed by plasmin. Kallikrein, T cell-associated serine proteinase, cathepsin B, cathepsin L, nerve growth factor-γ, human mast cell tryptase and prostate specific antigen are other proteinases that can also catalyze “*in vitro*” the conversion of pro-uPA [12]. uPA converts plasminogen to plasmin by specific cleavage of an Arg-Val bond in plasminogen. The interaction of uPA with its receptor uPAR is also important for cell migration.

The uPAR gene (Gene ID: 5329; http://www.ncbi.nlm.nih.gov/gene) encodes a single polypeptide chain of 313 aminoacid residues that after the maturation generates a 55-60kDa protein [21, 22]. The uPA/uPAR interaction on the cell surface induces conformational changes that facilitate the formation of a new complex with integrins and with several ECM proteins such as vitronectin [23, 24]. uPAR overexpression can also enhance growth factor activation and cell migration through the regulation of several pathways independent from its capacity of binding uPA and promoting plasmin activation [25].

**Figure 2:**
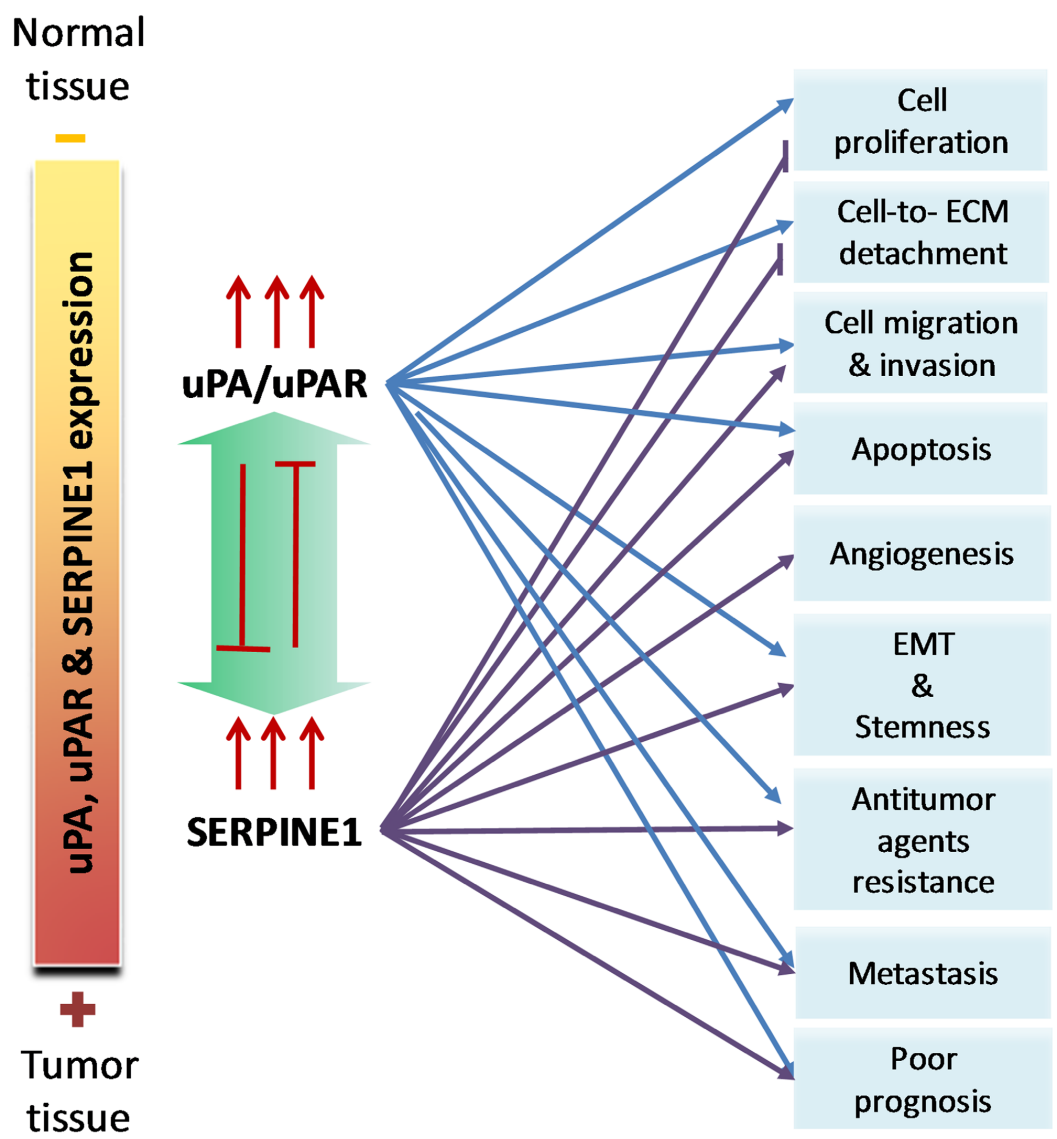
The pleiotropic effect of uPA/uPAR and SERPINE1 in head and neck squamous cell carcinoma

The uPA/uPAR complex expression plays a significant role on the invasive and metastatic potential of HNSCC [22, 26-28] (Figure 2). An active uPA increases the production of plasmin from plasminogen and this lead to ECM degradation, which in turn facilitates the invasion of cancer cells into the surrounding tissue, as well as their access to blood and lymph node vessels. α5β6 integrin can also activate uPA, which facilitates HNSCC cell motility [27, 29]. uPA and uPAR have been associated with an increase in the growth of head and neck cancer cells and with the activation of FAK and ERK1/2 signaling [30, 31]. Thombospondin has also been associated with the activation of uPA and cell invasion in HNSCC cells [32]. Plasmin cleaves a range of ECM proteins, such laminin and fibrin, and also activates metalloproteinases, such as the MMP2, previously associated with an increase of head and neck cancer cell invasion and metastasis [33-35]. Moreover, uPA and plasmin are involved in the activation of several growth factors, including HGF/SF and MSP, TGF-β, and FGF [12].

The expression of uPAR is associated with an invasive and metastatic phenotype in studies done using *in vivo* murine models of head and neck cancer. In oral squamous cell carcinoma xenografts, the inhibition of uPAR reduces tumor growth and downregulates the expression of genes previously associated with metastasis, such us MMP-2, MMP-9, VEGF-C, VEGF-D and VEGFR-3 [36]. A study conducted using an orthotopic murine model showed that the overexpression of uPAR in oral cancer cells generated infiltrative tumors with undefined margins and cytologic atypia [37]. These authors showed that the effect of uPAR on tumor cell invasion was associated with the activation of ERK1/2 MAP kinases and its co-localization with uPA and α3β1 integrin complex. uPAR can also promote the activation of the Ras-MAPK, Fak, Src and Rac and the PI3K-Akt pathways that have a significant effect on tumor cell migration [38]. Using an oral cancer metastatic mouse model, Zhang et al. showed that the expression of uPAR in cancer cells isolated from lymph node metastasis was higher than in cells isolated from primary tumor [39]. In nasopharyngeal carcinoma, a highly metastatic head and neck cancer [7], uPAR overexpression increases cell migration and invasion and promotes epithelial-to-mesenchymal transition and metastasis [25]. This process has been associated with the activation of the Jak-Stat pathway [40].

The inhibition of uPAR using antisense oligonucleotides reduces the invasiveness and the metastatic potential of head and neck cancer cells [41, 42].

In summary, most of the studies reported in head and neck cancer have shown that the overexpression of uPA/uPAR enhances tumor cell proliferation, migration and invasion. This effect is due to the activation of plasmin and ECM degradation, but it could also be the result of the indirect activation of several signaling pathways with a key role in tumor progression and metastasis, such as the PI3K-Akt pathway.

## SERPINE1 IN CELL PROLIFERATION, MIGRATION, INVASION AND METASTASIS

The SERPINE1 gene (encodes a clade E member of the serine protease inhibitor (SERPIN) superfamily that is the main regulator of the plasminogen activator system (PAs). SERPINE1 inhibits the urokinase-type plasminogen (uPA) and tissue-type plasminogen activator (tPA), which in turn, reduce the conversion of plasminogen to the active protease plasmin [21]. The SERPINE1 gene is located at 7q21.2-q22 and codifies for a single-chain glycoprotein of about 50kDa. SERPINE1 has several polymorphisms in the promoter region that are associated with gene transcription [43]. Its expression could also be modulated by several transcription factors such as SP1, AP1, SMAD proteins, TGF-β1, and p53 [44-46]. SERPINE1 expression could be epigenetically modulated [47, 48] and it has been described as a target for the miR-145 [49-51]. SERPINE1 expression is also related to the activation of hypoxia-related factors such as HIF-1[52]. The different protein conformations displayed by SERPINE1 are one of the particular features of this protein. Its active conformation inhibits tPA and uPA forming a complex with each enzyme, whereas its latent form does not react with their target proteinases [53]. A non-inhibitory substrate form of SERPINE1 that could be cleaved by PAs has also been described [54]. After the interaction between SERPINE1 and PAs, SERPINE1 is cleaved and acquires an inactive form. This is relevant because, depending on its conformation, SERPINE1 could interact with different proteins and activate distinct molecular pathways.

SERPINE1 is the main inhibitor of the uPA/uPAR complex, which induces its internalization through a process mediated by the lipoprotein receptor protein-1 (LRP1 receptor) [55]. Based on the pro-metastatic role of plasmin that promotes cell matrix degradation and cell migration, SERPINE1 expression would be expected to develop a protective effect against tumor dissemination throughout the inhibition of uPA/uPAR complex activity. However, most of the studies conducted to date, in several cancer types, indicate that SERPINE1 expression is associated with poor outcome and increased risk of metastasis [56]. This supports a multifunctional role for SERPINE1 that promotes tumor cell migration and metastasis through several pathways independent from PAs effectors. In this regard, SERPINE1 has more ligands than PAs, including ECM components, heparin and LRP1[57].

In normal and transformed human keratinocytes, SERPINE1 is up-regulated in response to EGFR and TGF-β and is associated with an increase in cell migration and invasion [58-60]. During the process of wound healing, TGF-β induces the expression of SERPINE1 in leading edge keratinocytes, which stimulates cell migration and re-epithelization [16].

SERPINE1 expression has also been associated with tumor cell migration and invasion in head and neck cancer cells [9, 61, 62]. We showed that the ectopic overexpression of SERPINE1 enhances head and neck cancer cell migration, and this is mediated by the phosphorylation and activation of Akt [62]. These findings are in agreement with several studies conducted in endothelial cells, fibrosarcoma and breast cancer cells indicating that SERPINE1 overexpression increases tumor cell migration and invasion through the activation of the PI3K-Akt pathway [63, 64]. SERPINE1 pro-migratory effect has been associated with LRP1 interaction, which in turn stimulates the Jak/Stat pathway [65, 66]. In thyroid cancer cells, LRP1 activates ERK and inhibits JNK-dependent pathways, which maintain the invasive capacity of tumor cells [65]. It would be interesting to evaluate the interaction of SERPINE1 and LRP1 and whether it has a similar effect on cell migration in head and neck cancer cells. Similar results have been observed using “*in vivo*” mice models. Bajou K et al. observed a decrease in local invasion and tumor vascularization of transplanted malignant keratinocytes in mice deficient in SERPINE1 expression. [67]. Invasion was restored by transfection with a vector expressing SERPINE1. SERPINE1 may also contribute to tumor aggressiveness by promoting tumor angiogenesis [67, 68][69].

The studies addressing the connection between SERPINE1 expression and cell proliferation have generated inconsistent results. Some groups showed that SERPINE1 expression increases cell proliferation, whereas others reported a reduction in cell proliferation [57, 70]. In head and neck cancer cells, we observed that the ectopic overexpression of SERPINE1 reduces cell proliferation, whereas its inhibition with shRNA increases cell proliferation [62].

## UPA/UPAR AND SERPINE1 IN APOPTOSIS REGULATION AND TUMOR RESISTANCE

Recent findings have suggested that in addition to its role in cell dissemination and metastasis, the expression of several components of the PA system could reduce the cytotoxic effect of anticancer drugs [71]. For instance, the uPAR expression has been associated with multi-drug resistance in small cell lung cancer cells [72]. uPA, uPAR, and SERPINE1 have also been associated with the efficacy of tamoxifen treatment in breast cancer [73, 74]. A high expression of these proteins increases drug resistance.

In head and neck and oesophageal cancer cells, uPA and SERPINE1 expression are upregulated after irradiation or reactive oxygen species exposure [75-79]. The activation of SERPINE1 is also associated with radiation resistance [80]. Moreover, the inhibition of uPAR has been associated with the downregulation of the multidrug resistance gene MDR1 [36]. Hypoxia is another factor that could contribute to tumor resistance. In this regard, the expression of SERPINE1 has been associated with the activation of hypoxia-related factors in head and neck cancer cells [52].

The plasminogen activator system proteins have been also associated with resistance to targeted therapies [71]. uPAR expression is associated with the development of resistance to EGFR inhibitor therapy in glioblastoma [81]. In head and neck cancer cells, the combination of uPAR down-regulation and EGFR inhibition showed a synergistic anti-tumor effect [82, 83]. Moreover, an association between the activation of EGFR signaling and SERPINE1 expression has also been described [60].

SERPINE1 (PAI-1) increases cisplatin resistance of head and neck tumor cells [62], while SERPINB2 (PAI-2) increases sensitivity [84]. Interestingly, both PA inhibitors showed an opposite effect on drug resistance. These findings and the fact that uPA/uPAR associates with resistance to therapy, suggest that the effect of SERPINE1 on tumor progression and drug resistance may be due to the activation of several signaling pathways that are independent from the inhibition of PA.

In the context of antitumor drug resistance, SERPINE1 is particularly interesting as it can enhance tumor progression through the inhibition of the apoptotic signaling. Thus, SERPINE1 over-expression has an anti-apoptotic effect [15, 59, 85, 86] that is mainly associated with the inhibition of Fas/Fas-L mediated apoptosis [68, 87]. SERPINE1 expression can also promote cell survival and block apoptosis by inhibiting caspase-3 activation [86]. Accordingly, we showed that the ectopic overexpression of SERPINE1 protects head and neck cancer cells from the apoptotic induction after cisplatin treatment [62]. This effect was mediated by PI3K-Akt pathway activation [62]. Similar findings have been reported in fibrosarcoma in which the expression of SERPINE1 protects cells from apoptosis through activation of the PI3K-Akt cell survival pathway [69, 88, 89].

On the basis of the foregoing, it may be concluded that SERPINE1 has an important role in protecting cells from apoptosis induction after the exposure to antitumor agents, and this should be considered when developing new treatment strategies, since it may be involved in cancer recurrence after therapy.

## UPA/UPAR AND SERPINE1, GO OR GROWTH EFFECT, EPITHELIAL-TO-MESENCHYMAL TRANSITION AND STEMNESS

The results reported to date suggest that SERPINE1 expression could increase cell migration and simultaneously reduce cell proliferation [62, 90]. In this regard, SERPINE1 could act as a switch between tumor cell proliferation and tumor cell migration [16, 90, 91]. This phenomenon commonly known as “go or growth” supports the notion that changes in tumor cell morphology associated with an increase in cell motility and migration, such us the cytoskeletal reorganization, are incompatible with enhanced cell proliferation [92]. Thus, tumor cells could activate the migration process to spread to other locations and once these cells reach a specific tissue, they could activate cell proliferation in order to colonize the tissue and generate metastasis. Cell migration is also accompanied by a reduction in apoptotic signaling that protects cells from death during their travel towards the target tissue. We observed that SERPINE1 inhibits apoptosis while reducing tumor cell proliferation, findings that are consistent with SERPINE1 enhancement of the invasive and migratory phenotype [62].

SERPINE1 is directly connected with the epithelial-mesenchymal transition (EMT) and the acquisition of stemness, two biological processes that are essential to control the transition from the proliferative to the invasive tumor phenotype [60, 93-97]. The EMT process is associated with the activation of the TGF-β pathway which also induces SERPINE1 activation [98]. In fact, SERPINE1 has been commonly used as a surrogate marker of EMT. EMT increases the plasticity of tumor cells and their capacity to spread to other tissues and it is closely linked to the acquisition of stem cell properties. In this sense, SERPINE1 overexpression has been observed in circulating tumor cells from breast cancer patients showing EMT-like features [97]. In head and neck tumors, Lee and colleagues showed that, SERPINE1 inhibition suppresses the self-renewal capacity of cancer stem cells through the inhibition of SOX2 [80]. The overexpression of SOX2, one of the main regulators of self-renewal in adult tissues, has also been associated with the development of different types of squamous cell carcinomas such as lung, esophagus and nasopharyngeal carcinomas [8, 99].

Recently, Yu et al. have shown a link between SERPINE1 expression and Notch signaling in differentiated thyroid cancer [100]. These authors showed that NOTCH1 induction downregulates SERPINE1 expression and it is associated with a reduction in lung metastasis development in mice. It would be interesting to explore if this effect is also present in HNSCC, as a high percentage of inactivating mutations in Notch has been observed in this tumor type [101, 102]. Notch signaling is a crucial regulator of stem cell renewal and promotes terminal differentiation of keratinocytes through the expression of p21 and caspase-3 [103-105].

In HNSCCs, the induction of EMT and the acquisition of cancer stem cell properties may partly account for the acquisition of resistance to antitumor agents after the PA system activation [80]. Changes in cell adhesion and cytoskeletal remodeling, experienced during the EMT-process, increase tumor cell plasticity and drug resistance, effects that correlate with an increase in the expression of uPAR and SERPINE1 [106, 107]. A mesenchymal-like phenotype showing stemness features has been observed in the most aggressive subtype of head and neck tumors that often overexpress SERPINE1 [108-111]. uPAR and uPA signaling could also contribute to cancer stemness, as it has been demonstrated in breast and pancreatic cancer cells [112, 113]. The high level of recurrence that occurs in nasopharyngeal carcinoma after chemoradiotherapy treatment [6] appears to be associated with the expression of EMT and cancer stem cell markers [8].

In summary, there is evidence in the literature supporting a role for SERPINE1 expression in the induction of EMT and the acquisition of stem cell properties, two key mechanisms for the generation of cancer stem cells, which are the transition from a proliferative to an invasive tumor phenotype and the development of antitumor resistance associated with late tumor recurrences.

## SERPINE1, UPA AND UPAR EXPRESSION AS PROGNOSTIC MARKERS IN HEAD AND NECK CANCER

SERPINE1 expression is higher in head and neck cancer tissue than in normal mucosa [27, 48, 62, 114-119]. Although a recent publication shows that SERPINE1 is up-regulated in cancer-associated fibroblasts and promotes the invasion of oral squamous cell carcinomas [120], immunohistochemical studies showed that the expression pattern of SERPINE1 in HNSCCs often differs from that observed in other cancer types. In head and neck tumors, SERPINE1 is expressed mainly in cancer cells, whereas in breast and colon cancers SERPINE1 is predominantly expressed in stromal cells rather than in cancer cells [116]. We observed, in this regard, that head and neck cancer cells showed membrane and cytoplasmatic positivity for SERPINE1 while tumor-adjacent normal tissue and stromal tissue areas were negative or showed negligible staining [62]. This could be particularly important as SERPINE1 could activate different biological pathways to promote tumor spread based on whether it is expressed in tumor cells or stromal cells.

**Figure 3:**
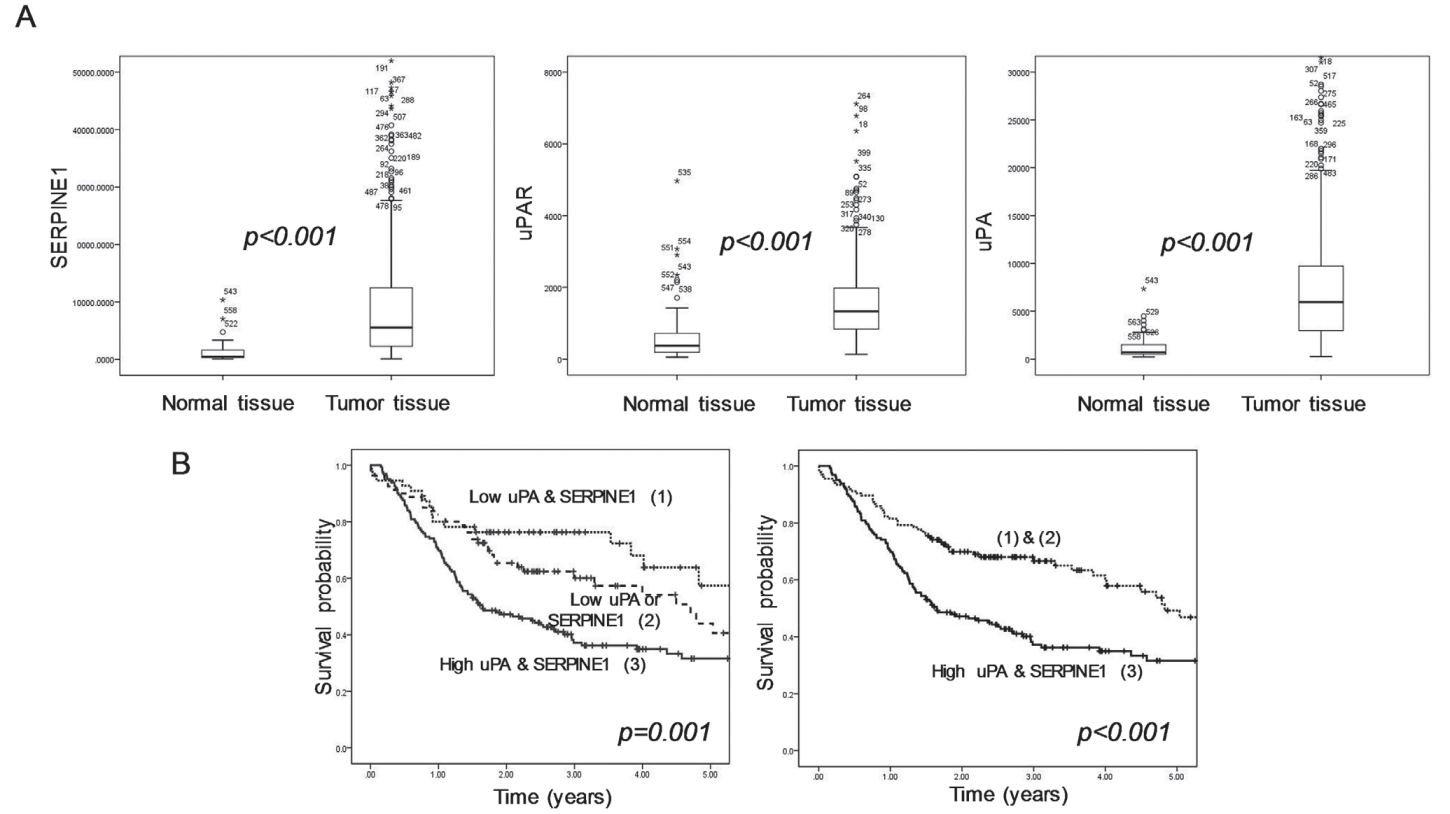
Expression profile of the uPA, uPAR and SERPINE1 genes in head and neck samples from HNSCC patients included in TCGA database **A.** Differences in gene expression between normal tissue (*n* = 44) and tumor tissue (*n* = 520) (Mann Whitney U test). **B.** Differences in survival between patients with tumors expressing high levels of uPA or SERPINE1 and patients with low tumor expression (log-rank test and Kapplan Meier curves. In order to perform the survival analysis, we selected patients with a minimal follow-up of 18 months (*n* = 297). All (unpublished) results shown are based upon RNA seq level 3 data generated by TCGA Research Network;http://cancergenome.nih.gov/.

In microarray gene expression studies, SERPINE1 expression has been identified as a HNSCC marker [109, 111, 121-123]. We used RNA seq level 3 data of 520 head and neck tumor samples and 44 normal tissue samples included in The Cancer Genome Atlas Database (TCGA) (http://cancergenome.nih.gov/) to study changes in uPA, uPAR and SERPINE1 expression after malignant transformation and its capacity to predict patient survival. We found overexpression of SERPINE1 in tumor tissue as compared to normal tissue (Figure 3A). Strojan et al showed that SERPINE1 expression was also higher in serum from patients with head and neck cancer than in healthy controls [124]. Lindberg et al showed that SERPINE1 expression was absent in normal, hyperplastic and dysplastic epithelia whereas a high SERPINE1 expression was present in incipient carcinoma and invasive carcinoma [116]. Additional large independent studies are needed to establish if the overexpression of SERPINE1 is also present in human papillomavirus (HPV) positive HNSCCs, a tumor subtype that differs in origin, biological features and clinical behavior from HPV negative tumors [125, 126].

A high expression of SERPINE1 in head and neck tumor biopsies has been associated with a poor clinical outcome [27, 61, 62, 116-118, 124, 127-131] (Table 1). Most of the studies conducted to date in HNSCC have concluded that patients with tumors showing a high SERPINE1 expression had a poorer disease-free or overall survival than patients with tumors expressing low levels. Besides its prognostic value, we also showed, by analyzing three independent cohorts of patients with HNSCC, that a high SERPINE1 expression increases the risk of metastasis after treatment [62].

**Table 1:** Expression of SERPINE1, uPA and uPAR as prognostic factors in head and neck cancer studies.

Study	uPA/uPAR/SERPINE1	Protein/RNA	Tumor vs Normal	Prognostic significance	Type	N
Pavon et al 2015 [62]	SERPINE1	Protein/RNA	Over-expressed	Poor prognosis	Re-/prospective	80 & 190
Pasini et al 2001 [117]	uPA/ SERPINE1	RNA	Over-expressed	------	Prospective	91
Speleman et al 2007 [118]	uPA/SERPINE1	Protein	Over-expressed	Poor prog. (SERPINE1)	Prospective	46
Yasuda et al 1997 [127]	uPA/SERPINE1	Protein	-----	-----	NA	28
Lindberg et al 2006 [116]	uPAR/SERPINE1	Protein	Over-expressed	-----	Retrospective	20
Nozaki et al 1998 [27]	uPA/uPAR/SERPINE1	Protein	-----	------	NA	34
Strojan et al 1998 [124]	uPA/SERPINE1	Protein	Over-expressed	-----	Prospective	58
Chin et al 2005 [128]	uPA/SERPINE1	Protein	Over-expressed	Poor prognosis	NA	62
Huang et al 2014 [84]	SERPINE1	Protein	Over-expressed	NS	Retrospective	43
Magnussen et al 2014[130]	uPAR/SERPINE1	Protein	Over-expressed	Poor prognosis	Retrospective	115
Dhanda et al 2014 [61]	SERPINE1	Protein	Over-expressed	Poor prognosis	Prospective	112
Hundsdorfer et al 2005 [131]	uPA/SERPINE1	Protein	Over-expressed	Poor prognosis	Prospective	79
Strojan et al 2000 [141]	uPA/SERPINE1	Protein	---	Poor prognosis (uPA)	NA	47
Yoshizawa et al 2011 [140]	uPA/uPAR	Protein	----	Poor prognosis	Retrospective	54

N:number of patients included in each study ; NS:non-significant; NA.: not available

However, the association between SERPINE1 expression and the clinicopathological characteristics has generated conflicting results. Some studies have shown that the SERPINE1 expression is higher in advanced stages [124, 127]. SERPINE1 expression has been also associated with the presence of lymph node metastasis and the perineural invasion [62, 118, 132]. Dhanda et al showed that SERPINE1 expression was higher in the invasive front and could predict the extracapsular spread in patients with oral cancer [61]. Once again, these findings support the association of SERPINE1 overexpression with an invasive and migratory tumor phenotype. Other authors did not find any association between the clinicopatological characteristics and SERPINE1 expression, probably due to the small sample size included in these studies [27, 129].

HNSCC studies showed that the expression of uPA and uPAR is also commonly higher in tumor tissue than in normal tissue [114, 115, 117, 119, 127, 133-137]. We also found similar results by analyzing the expression data (RNA seq level 3 data) of HNSCC samples included in TCGA project (Figure 3A) Tobacco smoke could induce the expression of uPA which is commonly overexpressed in premalignant and malignant lesions in the oral cavity [138]. Several studies showed that uPA or uPAR expression is associated with higher T stage, low grade of differentiation and the presence of lymph node metastasis [27, 117, 134, 139]. Most analyses conducted in head and neck cancer have concluded that the expression of uPA and uPAR is also associated with a poor clinical outcome [128, 140, 141] (Table 1).

SERPINB2, another component of the plasminogen activator system, has been also analyzed in patients with head and neck cancer, being associated with clinical outcome [84, 142]. In contrast to SERPINE1, SERPINB2 was identified as a favorable prognostic marker. Moreover, down-regulation was associated with a reduced overall survival in patients with HNSCC. The opposite effect on patient prognosis of both plasminogen inhibitors (SERPINE1 and SERPINB2) supports again the notion that SERPINE1 activates signaling pathways that are independent from its role as PA system inhibitors.

Taken together, the reported findings suggest that a high expression of the PA system components, especially of uPA and SERPINE1, is associated with a poor clinical outcome in patients with head and neck cancer. In this regard, our analysis of the gene expression profile of 297 tumors included in TCGA database with a minimum follow-up of 18 months, identified a subgroup of patients with a poor survival characterized by a high expression of uPA and SERPINE1 (Figure 3B). We observed that the expression of these proteins in pre-treatment tumor biopsies could be used to identify patients with a high probability of death and to distinguish them from patients with low risk. Many studies in breast cancer patients, have establish SERPINE1 and uPA as suitable markers for therapy decision-making in patients with early lymph-node negative breast cancer [143-146]. We expect that these markers will be increasingly studied in the near future to establish if they could also be used in HNSCC patients for helping treatment decision making, especially to identify those patients with a high risk of metastasis who could be treated with adjuvant chemotherapy or chemo-radiation.

## INHIBITION OF THE PLASMINOGEN ACTIVATOR COMPONENTS AS A THERAPEUTIC STRATEGY IN HNSCC

The reported results point out that the components of the PA system uPA, uPAR and SERPINE1 could be good targets for HNSCC therapy, so that their inhibition could represent a relevant strategy to increase the efficacy of current antitumor agents. In this sense, the antitumor activity of several small molecules inhibitors of SERPINE1, initially developed as antithrombotic agents, is currently being evaluated [87, 147-150]. These specific inhibitors usually block the interaction between SERPINE1 and uPA and generate conformational changes that result in the irreversible conversion of SERPINE1 into its latent or cleaved forms [15, 147, 151-153].

*In vitro* and *in vivo* xenografts models have shown an effect for these inhibitors on angiogenesis, apoptosis induction and tumor growth [87, 154-158]. In pre-clinical models, Tiplaxtinin, one of the most studied SERPINE1 inhibitors, is able to block the growth and induce apoptosis in bladder carcinoma, fibrosarcoma and head and neck cancer cells [80, 87, 156]. However, additional preclinical studies and subsequent clinical trials are necessary to show if these specific inhibitors could be used as a targeted therapy in HNSCCs patients whose tumors overexpress SERPINE1. Inhibitors of uPAR or uPA are also being developed and tested as antitumor agents in patients with breast, pancreatic and head and neck cancer in phase I-II trials [46, 159-162].

Moreover, as the activation of the PI3K-Akt pathway was commonly associated with SERPINE1 overexpression, the use of new inhibitors of PI3K-Akt pathway, currently under clinical investigation, could also be considered as an option to treat tumors overexpressing SERPINE1.

## CONCLUSIONS AND PERSPECTIVES

In summary, the overexpression of uPA/uPAR enhances tumor cell proliferation, migration and invasion and plays a key role in metastasis development, conferring poor prognosis. This system appears to act mainly by activation of plasmin, involved in ECM degradation, and through the activation of several signaling pathways such as the PI3K-Akt pathway. SERPINE1 overexpression also enhances tumor cell migration and metastasis dissemination, promotes angiogenesis, protects cells from Fas/Fas-L mediated apoptosis and is associated with poor prognosis. The fact that the overexpression of uPA/uPAR and its main inhibitor SERPINE1, produce similar effects on cell migration, tumor spread and prognosis may seem contradictory, but several reports suggest that SERPINE1 activates signaling pathways independent of the inhibition of the uPA/uPAR complex. Both, uPA/uPAR and SERPINE1 are closely associated with the induction of EMT and the acquisition of cancer stem cell properties, which could contribute to resistance to therapy.

uPA/uPAR and SERPINE1 may be useful as prognostic biomarkers, since they are commonly overexpressed in HNSCCs and are associated with a poor clinical outcome. The determination of these markers in pre-treatment tumor biopsies could be used to stratify patients according to their risk of metastasis development. In the future, these markers, especially uPA and SERPINE1, could be used for treatment decision making to identify patients with a high risk of metastasis development, who could benefit from adjuvant chemotherapy or chemo-radiotherapy. However, they should be validated in independent clinical trials in order to clarify their clinical value and to identify suitable parameters for their detection and patient stratification. We also expect in the future that the specific inhibitors of uPA/uPAR and SERPINE1, already in clinical trials, could be tested in HNSCCs in combination with other drugs or radiation in an attempt to improve current antitumor therapy. The inhibition of the PA system could be particularly relevant to reduce lymph node recurrences and metastatic dissemination, one of the major challenges to prevent deaths for head and neck cancer.
